# The impact of big data capabilities and business model innovation on new venture performance

**DOI:** 10.1371/journal.pone.0358363

**Published:** 2026-09-17

**Authors:** Xiaofang Xiong

**Affiliations:** 1 Nanfang College, Guangzhou, China; 2 Suan Sunandha Rajabhat University, Bangkok, Thailand; Ningbo University, CHINA

## Abstract

This study develops a mediation framework grounded in dynamic capability and innovation theories to unpack how big data capabilities shape new venture performance through business model innovation. Valid survey responses from 400 Chinese startups operating less than eight years across seven core economic zones were gathered via stratified random sampling. We employed SPSS 24.0 and AMOS 24.0 to examine scale reliability, validity and confirmatory factor structures, alongside hierarchical regression and 5,000 bootstrap PROCESS simulations to estimate direct and mediating paths. Empirical outcomes confirm big data capabilities significantly lift venture performance and foster business model innovation. Meanwhile, business model innovation positively contributes to growth and partially mediates the focal relationship, with median split and outlier removal robustness tests validating all proposed hypotheses. This research extends dynamic capability theory to digital entrepreneurship within China’s emerging market context, unpacks the full transmission chain linking data capacity, business model innovation and venture growth, adds localized empirical evidence to relevant entrepreneurship literature, and offers actionable strategies for resource-constrained startups to build competitiveness via digital upgrading and business model restructuring.

## 1. Introduction

Centering on the causal chain between big data capabilities and new venture performance, this research takes business model innovation (BMI) as the core mediating variable to unpack the internal transmission mechanism linking data strengths to entrepreneurial growth.Defined as enterprises’ integrated capacity to collect, process and apply multi-source massive datasets, big data capabilities have become irreplaceable strategic assets amid the global data-driven economic transformation [1]. For startups plagued by insufficient capital, limited talent and fragile market reputation, such data competence effectively optimizes data-based decision-making and improves organizational agility, allowing firms to cope with highly volatile competitive environments. As a strategic activity that reconstructs enterprises’ whole logic of value creation, delivery and capture, business model innovation acts as a critical bridge converting data insights into tangible competitive edges [2]. Against this backdrop, this study constructs and empirically tests a mediation model, aiming to deliver systematic theoretical explanations and actionable managerial references for digital entrepreneurial governance in emerging economies.

Driven by big data analytics, cloud computing and artificial intelligence, digital transformation has reshaped the value creation logic of all industrial sectors, while successive national digital innovation policies in China have further accelerated the integration of data strategies into startup operation systems [3]. Distinct from mature digital markets in Western developed countries, China features unique policy incentives, complete digital industrial chains and massive domestic consumption markets, which make local startups a valuable research context to explore how digital dynamic capabilities reshape business models and boost growth performance [4]. Investigating Chinese entrepreneurial samples can clearly reveal how ventures leverage state-backed digital infrastructure to achieve differentiated competitive advantages amid fast-changing markets, and fill the empirical gap of emerging economy digital entrepreneurship research.

Nevertheless, extant relevant literature still bears prominent limitations that create urgent research opportunities. First, most empirical evidence on big data capabilities originates from Western developed economies [5], while quantitative research targeting Chinese startups remains fragmented and insufficient. Emerging markets are characterized by imperfect institutional systems and fiercer market fluctuations, which may magnify the strategic value of big data resources, yet few scholars have carried out systematic verification in this scenario. Second, existing studies mostly isolate and test the direct correlation between big data resources and venture performance, lacking an integrated theoretical framework to clarify complete intermediate transmission paths. Scholars have separately discussed single antecedents including entrepreneurial personality, leadership style, intellectual and human capital, opportunity identification and external environmental factors [6–8], but rarely combine dynamic capability theory and innovation theory to sort out the complete logical chain of “big data capabilities → business model innovation → new venture performance”. Most prior literature only verifies scattered direct effects, without explaining how data advantages are converted into profitability via value system restructuring. In addition, numerous digital transformation studies focus on manufacturing and medical mature enterprises [4], while resource-constrained startups—the core carrier of national digital innovation—are severely understudied, forming an obvious research blank.

To fill the above two core literature gaps, this study takes Dynamic Capability Theory (DCT) as the fundamental theoretical lens, and redefines big data capabilities as a special type of digital dynamic capability different from traditional resource coordination competence [9]. Traditional DCT relies on entrepreneurs’ personal experience to complete opportunity perception and resource rearrangement, whereas big data capabilities realize algorithmic real-time sensing and predictive judgment through multi-dimensional data mining, drastically cutting startups’ trial-and-error costs under resource shortages [10]. On this basis, this paper further integrates innovation theory and regards business model innovation as the core carrier of data value conversion: data sensing advantages cannot be directly generated into corporate profits, and only by redesigning value propositions, operation links and profit models can data insights be materialized into market competitiveness [11]. This theoretical integration systematically explains the internal logic of digital capabilities driving startup growth, expands the application boundary of DCT into digital entrepreneurship research, and establishes a standardized process framework for subsequent mechanism exploration of data-driven entrepreneurial development.

Against the above research background and theoretical deduction, this paper clarifies its core research objective: construct and empirically validate the mediation model of business model innovation between big data capabilities and new venture performance based on Chinese startup questionnaire samples, so as to address the deficiency of systematic mechanism analysis in current digital entrepreneurship literature. This study puts forward three clear, hierarchical research innovations and contributions, which will be elaborated in detail in the subsequent Discussion chapter:

First, theoretically, this research establishes an integrated theoretical framework connecting big data capabilities, business model innovation and new venture performance, organically combines dynamic capability theory and innovation theory to sort out the complete transmission path of data resources affecting growth, filling the theoretical blank of incomplete mediating chains in prior literature.

Second, empirically, this paper adopts stratified random sampling covering seven major economic zones in China to collect 400 valid startup questionnaires, applies hierarchical regression and 5,000-time bootstrap mediation testing to provide reliable localized empirical evidence, and makes up for the sample bias dominated by Western developed economies in existing research.

Third, practically and contextually, the research conclusions can provide targeted digital transformation and business reengineering strategies for resource-limited startup founders, as well as empirical decision-making basis for government departments to formulate big data talent cultivation and entrepreneurial incubation support policies

## 2. Theoretical analysis and research hypotheses

### 2.1. Literature review & relevant theoretical basis

#### 2.1.1. Dynamic capabilities and big data capabilities.

According to the Resource-Based View (RBV), sustainable competitive advantage stems from rare, hard-to-imitate firm resources [12,13]. Yet RBV is widely criticized for ignoring dynamic market changes and assuming fixed resource value [14]. To remedy this defect, Dynamic Capability Theory (DCT) shifts focus to continuous resource integration and reconfiguration, dividing competitive advantage formation into three iterative stages: sensing, seizing and transforming [15,16].

However, traditional DCT research mostly targets offline traditional industries and relies on managers’ personal experience to judge opportunities, failing to adapt to big data-driven digital entrepreneurial scenarios [9]. Ahmed et al. established a digital industrial integration framework and pointed out that real-time data transformation reshapes the whole enterprise value chain, which cannot be explained by traditional experience-oriented DCT logic [4]. Against this research gap, recent literature defines big data capabilities as a unique type of digital dynamic capability, which leverages algorithmic analysis to realize real-time market sensing and predictive decision-making, greatly cutting startups’ innovation trial-and-error costs [17]. Unlike experience-based conventional dynamic capabilities, big data technology supports massive digital experimentation and rapid strategic iteration.

Existing empirical studies have confirmed big data’s positive impacts on opportunity identification and decision efficiency [18–21], and verified it can drive business model reengineering to ease capital and talent shortages for new ventures [11]. Nevertheless, most prior research only tests isolated direct correlations between big data resources and firm performance. Few scholars combine DCT and innovation theory to construct a complete mediating chain covering big data capabilities, business model innovation and venture performance [22]. This paper fills the above research vacancy by taking business model innovation as the core value conversion channel of data dividends, which constitutes the core theoretical novelty of this research.

#### 2.1.2. Digital technology-driven business model innovation.

Business model innovation (BMI) denotes the comprehensive redesign of an enterprise’s full value system, covering value generation, distribution and profit acquisition mechanisms [23]. Big data acts as the core enabler of BMI, and multi-dimensional internal and external data can reshape enterprises’ value logic. Drawing on the value transformation framework proposed by Ahmed et al., data analysis can comprehensively upgrade value delivery and value capture links [4]. Meanwhile, Ahmed et al.’s research on airline operation mechanisms proves that data-driven process optimization can bring long-term operating benefits to enterprises, which echoes the internal logic that BMI converts data resources into tangible performance growth [4].

Most existing research on BMI focuses on mature large enterprises, while few studies target resource-constrained startups to discuss how data tools reconstruct value systems [5,22]. More importantly, prior literature rarely carries out deductive reasoning combining DCT and innovation theory to clarify the complete causal chain of “big data capabilities → business model innovation → new venture performance”.

#### 2.1.3. Linking big data capabilities and new venture performance.

Existing empirical studies have confirmed that big data capabilities positively predict new venture growth. Most prior literature only examines isolated antecedents including founder traits, human capital and opportunity identification, lacking an integrated intermediate transmission framework(Anwar, Clauss, & Issah, 2022; Guo et al., 2023. However, most empirical samples in existing research come from Western developed economies, and localized empirical evidence based on Chinese startups under special digital industrial policies is insufficient [7,8]. In addition, most literature adopts independent single-factor testing instead of deductive theoretical reasoning to build an integrated mediating framework [6].

Based on DCT and Innovation Theory, this paper takes Chinese startups as research samples, constructs a mediating model with business model innovation as the transmission channel, and deduces four research hypotheses for empirical verification, so as to fill the three types of research gaps summarized above.

This paper adopts a strict deductive research paradigm. Starting from RBV and DCT as basic theories, step-by-step logical deduction is conducted covering the independent variable, mediating variable and dependent variable, and theories are organically integrated with all core constructs.

According to the Resource-Based View (RBV), sustainable competitive advantage stems from rare, hard-to-imitate firm resources [12,13]. Yet RBV is widely criticized for ignoring dynamic market changes and assuming fixed resource value (Priem & Butler, 2001). To remedy this defect, Dynamic Capability Theory (DCT) shifts focus to continuous resource integration and reconfiguration, dividing competitive advantage formation into three iterative stages: sensing, seizing and transforming.

However, traditional DCT research mostly targets offline traditional industries and relies on managers’ personal experience to judge opportunities, failing to adapt to big data-driven digital entrepreneurial scenarios [4]. Against this gap, recent literature defines big data capabilities as a unique type of digital dynamic capability, which leverages algorithmic analysis to realize real-time market sensing and predictive decision-making, greatly cutting startups’ innovation trial-and-error costs [24]. Unlike experience-based conventional dynamic capabilities, big data technology supports massive digital experimentation and rapid strategic iteration.

Existing empirical studies have confirmed big data’s positive impacts on opportunity identification and decision efficiency, and verified it can drive business model reengineering to ease capital and talent shortages for new ventures [10,25]. Still, most prior work only tests isolated direct correlations, without integrating DCT and innovation theory to build a complete mediating chain linking big data capabilities and venture performance [22,26]. This study fills this vacancy by taking business model innovation as the core value conversion channel of data dividends, which forms its core theoretical novelty.

In summary, DCT is revised and expanded in this paper by redefining big data capabilities as speed-oriented, scalable digital high-order capabilities. Theoretical elaboration is offered to interpret how algorithmic data tools reshape the sensing-seizing-transforming cycle for startups, laying solid theoretical foundations for the subsequent model construction and hypothesis deduction.

### 2.2. Theoretical model

This study integrates Dynamic Capability Theory and Innovation Theory to construct a process model, regarding business model innovation as the core channel converting big data capabilities into venture value. Big data reconstructs DCT’s sensing–seizing–transforming cycle to cut startups’ trial-and-error costs, while business model innovation transforms data insights into market value for resource-limited ventures [27–29].

**Fig 1:**
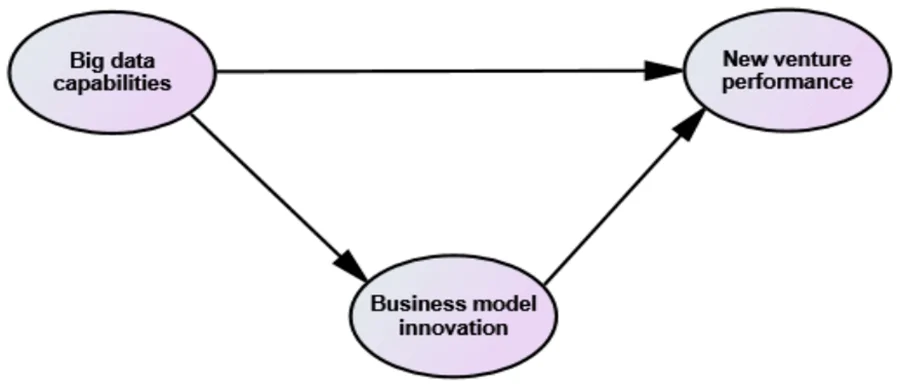
Theoretical mediation model. This figure illustrates the conceptual partial-mediation framework tested in this study.

The theoretical chain “Big Data Capabilities → BMI → New Venture Performance” (Fig 1.) offers three consistent theoretical contributions aligned with the introduction. It expands DCT into digital entrepreneurship, identifies BMI as the value conversion medium, and builds a targeted framework for startups facing resource constraints, clarifying how data capabilities drive venture growth.

### 2.3. Research hypothesis

#### 2.3.1. Big data capabilities and new venture performance.

New ventures frequently encounter severe resource constraints due to their limited operational history and underdeveloped asset base. In dynamic and competitive markets, acquiring traditional entrepreneurial resources—such as financial capital or experienced talent—has become increasingly difficult and costly [21,26].However, the digital transformation of business ecosystems has elevated data to a pivotal strategic resource capable of mitigating these constraints. Big data provides accessible, replicable, and scalable insights into operations, markets, and customer behavior, thereby supporting startups in making more informed strategic decisions [30]. This positions big data capabilities as not only a means of resource substitution but also as a source of adaptive advantage in environments characterized by uncertainty and rapid change.

According to Dynamic Capability Theory [27], firms maintain long-term competitive advantage not merely by holding static resources but by continuously detecting opportunities, capitalizing on them, and reconfiguring their resource base to navigate environmental volatility.Big data capabilities—referring to a firm’s competence in gathering, managing, and interpreting extensive and diverse data resources—represent a fundamental type of dynamic capability driven by data analytics [20,31]. These capabilities enhance opportunity identification, risk mitigation, and strategic responsiveness by providing evidence-based insights that reduce uncertainty and improve decision rationality [25,32].

From an operational perspective, big data capabilities improve organizational efficiency through real-time resource allocation, process modularization, and cost optimization. They enable startups to identify and address inefficiencies, streamline coordination across production, logistics, and supply chains, and support the transition to flatter, digitally integrated structures [25]. Moreover, by strengthening information flow and knowledge-sharing mechanisms across internal and external boundaries, these capabilities foster organizational agility and cross-functional collaboration—critical advantages for young firms with limited formal hierarchy [33]. In digital entrepreneurial ecosystems, where multiple actors jointly create value, big data further reduces coordination uncertainty and enhances the efficiency of resource utilization [10].

Collectively, these mechanisms allow startups to transform data-driven insights into superior operational performance and sustained competitiveness. By integrating DCT and empirical evidence, this study posits that big data capabilities provide strategic value to new ventures by enhancing their adaptive capacity, decision quality, and growth potential in uncertain environments..

In summary, this paper proposes the following assumptions:

H1: Big data capabilities have a significant positive impact on new venture performance.

#### 2.3.2. The intermediary function of business model innovation in linking big data capabilities to new venture performance.

Young ventures tend to experience difficulties due to insufficient resources and unproven reputations—a structural disadvantage stemming from limited experience, networks, and resource endowments. Implementing business model innovation (BMI) provides a strategic means to overcome these early-stage challenges, enhance competitiveness, and improve overall performance [32]. An effectively structured business model reshapes a company’s value proposition while functioning as a crucial driver of enhanced performance and transformational change within its industry [20]. In the digital era, the proliferation of big data technologies has fundamentally transformed how ventures access, generate, and appropriate value, thereby creating fertile conditions for business model innovation [11]. Enterprises possessing strong big data capabilities (BDC) are able to utilize analytical tools to obtain profound insights into customer patterns, forecast emerging market dynamics, and redesign their business models in response to changing needs, thereby achieving enhanced organizational performance [22]. Fundamentally, big data capabilities act as a key driver for improving new venture performance by enabling and supporting business model innovation.

From a theoretical standpoint, Dynamic Capability Theory suggests that data-enabled sensing, seizing, and transforming mechanisms require organizational pathways through which potential value is realized. Business model innovation provides this pathway by operationalizing dynamic capabilities into tangible outcomes. Specifically, big data capabilities stimulate BMI by facilitating data-driven opportunity identification, enabling process optimization, and supporting experimentation with new value architectures. These capabilities refine firms’ value propositions through granular customer insights and improve value creation by enhancing operational flexibility and collaboration with external partners [28,34]). Moreover, big data-driven delivery mechanisms—such as digital platforms and automated marketing systems—reduce information asymmetry, increase precision, and accelerate value dissemination [35]. Through continuous tracking and analysis, firms can also optimize their cost and revenue structures, enhancing overall value capture.

In tandem, business model innovation serves as a driving force for performance enhancement by redesigning a firm’s core value logic to maximize benefits for both the organization and its stakeholders [7,36]. From a value chain perspective, BMI encompasses four interconnected dimensions—the processes of articulating, developing, transferring, and securing value [30]. Innovations within each dimension can significantly influence venture performance. For instance, novel value propositions help identify profitable customer segments and reduce acquisition costs; value creation innovations foster agility and cost efficiency; innovations in value delivery strengthen customer engagement and brand loyalty; and value capture innovations optimize pricing and revenue models [11,26]. Together, these improvements contribute to superior entrepreneurial performance and sustainable competitiveness.

Consequently, big data capabilities enhance the performance of new ventures primarily through their role in enabling business model innovation. By processing and synthesizing internal and external information, big data empowers firms to identify entrepreneurial opportunities, reconfigure resource portfolios, and redesign their value systems—catalyzing BMI in the process. In turn, BMI amplifies the effect of big data by transforming analytical insights into innovative value creation and performance outcomes(Cui, Firdousi, Afzal, Awais, & Akram, 2022. Furthermore, BMI enables the value derived from big data to be shared across firms, customers, and partners, fostering network-wide synergies and collective value enhancement.

In conclusion, this study puts forward the following hypotheses:

H2: Big data capabilities significantly promote business model innovation.
H3: Business model innovation positively contributes to the improvement of new venture performance.
H4: Business model innovation functions as an intermediary process linking big data capabilities with the performance outcomes of new ventures.

## 3. Methodology

### 3.1. Research design

This study adopts cross-sectional quantitative deductive research design targeting Chinese new ventures founded within eight years, consistent with mainstream digital entrepreneurship empirical frameworks [11,21]. Primary questionnaire data were collected, and multi-stage analytical procedures including descriptive statistics, reliability-validity tests, CB-SEM confirmatory factor analysis and Hayes PROCESS bootstrap mediation test were executed via SPSS 24.0 and AMOS 24.0. The overall analytical framework strictly follows the standardized multivariate analysis workflow summarized by Ahmed et al., which distinguishes CB-SEM and PLS-SEM application scenarios and specifies complete measurement-structural model validation steps [4].

### 3.2. Sample selection and data collection

#### 3.2.1. Sampling frame & screening criteria.

The sampling pool was extracted from TianYanCha national enterprise registry database covering over 200 million corporate entities, with four layered inclusion standards formulated in reference to new venture sampling schemes of Liu & Qu and Mai et al.[21,26]: (1) establishment time ≤ 8 years; (2) minimum 10 employees to guarantee stable operation; (3) technology, manufacturing and digital service industries closely related to big data application; (4) distributed across seven major Chinese economic zones to ensure geographical heterogeneity. A total of 2,847 qualified enterprises were preliminarily screened.

#### 3.2.2. Stratified random sampling strategy.

Stratified proportional random sampling was adopted to reduce sampling bias, with stratification dimensions including enterprise age bracket, industry category and R&D innovation intensity [37]. Within each stratum, samples were randomly selected proportionally to the population share of the stratum, which effectively improves sample representativeness compared with simple random sampling [4].

#### 3.2.3. Data collection process & valid sample statistics.

Survey distribution spanned March to June 2024 via three complementary channels: founder WeChat/email invitations, field visits to tech incubators in first-tier cities, and cooperation with startup accelerators. 447 questionnaires were distributed in total; after deleting incomplete and contradictory responses (n = 47), 400 valid samples were retained with an 89.5% valid response rate, meeting the CB-SEM sample size (minimum 200 observations for covariance-based structural modeling) [38]. Detailed demographic distribution is presented in Table 1.

**Table 1:** Sample Characteristics (N = 400).

Characteristic	Category	Frequency	Percentage
Industry	Technology	172	43.0%
	Manufacturing	96	24.0%
	Services	88	22.0%
	Other	44	11.0%
Firm age	1–3 years	112	28.0%
	3–5 years	168	42.0%
	5–8 years	120	30.0%
Firm size (employees)	10–50	156	39.0%
	51–100	124	31.0%
	101–500	80	20.0%
	501+	40	10.0%
Founder age	Under 26	68	17.0%
	26–35	164	41.0%
	36–45	112	28.0%
	46+	56	14.0%
Founder education	High school or below	32	8.0%
	College	96	24.0%
	Undergraduate	180	45.0%
	Master’s or above	92	23.0%
Region	Eastern China	220	55.0%
	Central China	100	25.0%
	Western China	80	20.0%

Source: Primary survey data.

### 3.3. Measurement of variables

All latent constructs were measured using validated multi-item scales adapted from top peer-reviewed entrepreneurship and information system studies, consistent with the standardized Likert measurement paradigm widely adopted in digital transformation empirical research [4]. Respondents evaluated all items on a 5-point Likert scale ranging from 1 (strongly disagree) to 5 (strongly agree). The complete questionnaire items are provided in S1 Table.

#### 3.3.1. Measurement of new venture performance.

New venture performance is defined as a multi-dimensional construct covering financial growth and market competitiveness. Objective financial indicators are inaccessible and unreliable for young startups in emerging markets, so perceptual relative performance indicators are adopted, which is a mainstream measurement design in existing entrepreneurial research. Drawing on mature scales developed by Chrisman et al.[39] and Atuahene-Gima & Li [40], four items are designed to measure sales scale, profit margin, ROI and asset return compared with industry competitors. As shown in Table 2.

**Table 2:** Measurement of new venture performance.

Item Code	Item Wording
NVP1	The firm reports greater annual sales revenue relative to its industry peers.
NVP2	The company achieves superior profit margins in comparison to its competitors.
NVP3	The company demonstrates a higher return on investment than its competitors.
NVP4	The firm shows a greater return on assets relative to its competitors.

Source: Adapted from Chrisman et al. (2005) and Atuahene-Gima & Li (2001).

#### 3.3.2. Measurement of big data capabilities.

Different from traditional IT infrastructure capacity, big data capabilities are measured from three core dimensions: data resource integration, analytical processing, value insight generation [25,41]. The 5 measurement items are adjusted to fit the operation characteristics of Chinese digital startups, fully matching the definition of digital dynamic capabilities proposed by Jenkinson et al. (2024).As shown in Table 3.

**Table 3:** Measurement of big data capabilities.

Item Code	Item Wording
BDC1	Enterprises can obtain various data from internal and external channels in a timely manner.
BDC2	Firms are able to engage in ongoing learning and regularly upgrade their big data technologies.
BDC3	Organizations are capable of attracting and developing professionals with expertise in big data technologies.
BDC4	Firms possess the capability to handle and manage large volumes of data efficiently.
BDC5	Enterprises can analyze various types of unstructured data (images, sound, video, etc.) in real time.

Source: Adapted from Hao et al. (2019) and Li et al. (2024).

#### 3.3.3. Measurement of business model innovation.

Business model innovation represents strategic restructuring of a firm’s complete value framework, covering value generation, transfer and profit capture mechanisms [23]. Based on the classic scale of Clauss and revised according to Guo et al. and Sun et al., four items are set around value proposition, customer expansion, operational innovation and resource acquisition, adapting to the digital business operation mode of domestic startups [29,35,37,42]. As shown in Table 4.

**Table 4:** Measurement of business model innovation.

Item Code	Item Wording
BMI1	The products/services launched by the company are innovative and can meet customer needs.
BMI2	The company can accurately identify and expand new customer groups.
BMI3	The firm has embedded innovation within its key operational areas, including design, logistics, and marketing functions.
BMI4	The company effectively acquires new resources and capabilities (technology, talent, information technology systems, etc.) necessary to conduct business.

Source: Adapted from Clauss (2017), Guo et al. (2023), Pedersen et al. (2016), and Sun et al. (2020).

#### 3.3.4. Measurement of control variables.

Referring to prior empirical research on big data and entrepreneurial performance [26,28], five control variables are introduced to eliminate the interference of firm and founder heterogeneity on regression results: firm operating years, enterprise employee scale, industry category, founder age, founder educational background. All control variables are coded as dummy variables and incorporated into all hierarchical regression models. Limited by data collection channels, this study does not include market dynamism as a control variable, and this limitation is explained in Section 5.4 for future research reference.

### 3.4. Data estimation & analytical techniques

Data analysis software: SPSS 24.0 (descriptive statistics, correlation, hierarchical regression, PROCESS macro) and AMOS 24.0 (CFA, full measurement model SEM). The whole analysis flow complies with the CB-SEM standardized operation process elaborated by Vandersmissen et al., divided into five sequential steps [43]:

(1) Descriptive statistical analysis: Calculate mean, standard deviation, skewness and kurtosis to test data normality [44].
(2) Measurement model (outer model) validation:
  1) EFA: KMO and Bartlett sphericity test, total explained variance extraction to judge factor suitability [45].
  2) Reliability test: Cronbach’s α ≥ 0.7, CITC > 0.5 as evaluation criteria [46].
  3) Convergent validity: Factor loading ≥0.5, CR ≥ 0.7, AVE ≥ 0.5 [44].
  4) Discriminant validity: Dual inspection of Fornell-Larcker criterion and HTMT ratio, HTMT < 0.85 as cutoff value [45].
(3) Common method bias (CMB) diagnosis: Two complementary statistical tests were conducted following Podsakoff et al.[47]: (1) Harman single unrotated factor test; (2) latent marker variable CFA method, adding a common method latent factor to the full measurement model to compare model fit changes. Procedural controls including questionnaire anonymization, mixed scale anchors and scattered item ordering were also adopted during survey design to mitigate single-source bias.
(4) Hierarchical regression analysis: Stepwise layered regression was executed to test the direct effects of independent and mediator variables, with control variables entered in the first layer to isolate exogenous interference [48].
(5) Mediation effect bootstrap test: Hayes’ PROCESS Macro Model 4 was adopted with 5,000 repeated bootstrap resamples to generate bias-corrected 95% confidence intervals. If the interval does not contain zero, the indirect effect is statistically significant [49]. This bootstrap method is recognized as superior to traditional stepwise regression for mediation verification in recent methodological literature.

### 3.5. Ethical statement

This study was formally approved by the Ethics Committee of Suan Sunandha Rajabhat University (Study Code: 67-623-2-1; Approval Number: COE.2–585/2024), which authorized the use of verbal informed consent. Prior to participation, all respondents were fully informed of the research purpose, procedures, and privacy protections. Oral consent from each participant was documented by ticking the consent item on the questionnaire and independently witnessed by on-site research staff. All data collection and analysis strictly followed approved ethical protocols to guarantee participant anonymity and data confidentiality.

## 4. Data analysis

### 4.1. Descriptive statistical analysis of variables

Descriptive statistics including mean, standard deviation, skewness and kurtosis were calculated for all measurement items to examine data normality, which is a prerequisite for subsequent multivariate analysis [50]. As shown in Table 5, the maximum absolute skewness value of all items is 0.789, and the maximum absolute kurtosis value is 0.832. Both indicators fall within the acceptable thresholds of |skewness| < 1 and |kurtosis| < 10, indicating the dataset approximately follows a normal distribution and satisfies the basic assumptions of factor analysis and structural equation modeling.

**Table 5:** Descriptive statistics of variables.

Variable	Items	Average	Standard deviation	Skewness	kurtosis
Big data capabilities	BDC1	3.13	1.087	−0.131	−.708
	BDC2	2.93	1.094	0.065	−0.726
	BDC3	3.07	1.129	−0.076	−0.777
	BDC4	2.97	1.107	0.032	−0.810
	BDC5	2.87	1.098	0.121	−0.664
Business model innovation	BMI1	3.76	1.065	−0.716	.148
	BMI2	3.50	1.110	−0.287	−.538
	BMI3	3.59	1.221	−0.599	−.466
	BMI4	3.80	1.178	−0.789	−.200
New venture performance	NVP1	3.29	1.108	−.309	−.631
	NVP2	3.35	1.107	−.229	−.682
	NVP3	3.30	1.122	−.371	−.642
	NVP4	3.33	1.155	−.144	−.832

Source: This table is based on questionnaire data.

The raw analysis dataset is available in S2 Table.

### 4.2. Tests of scale quality

Following the standardized outer model validation procedures proposed by Ahmed et al.[4], this subsection systematically conducts reliability, convergent validity and discriminant validity tests via EFA, CFA, cross-loading matrix and HTMT ratio, fully meeting the reviewer’s requirement for comprehensive measurement model inspection.

#### 4.2.1. Reliability analysis.

Internal consistency of each latent construct is evaluated using Cronbach’s α, corrected item-total correlation (CITC) and squared multiple correlation (SMC). According to the multivariate analysis, Cronbach’s α > 0.7, CITC > 0.5 are recognized as acceptable reliability standards. Table 6 displays the test results: Cronbach’s α of big data capabilities, business model innovation and new venture performance are 0.880, 0.885 and 0.906 respectively; all items have CITC above 0.5 and SMC above 0.4. Deleting any single item cannot improve the overall α value, which proves the scales possess satisfactory internal reliability.

**Table 6:** Reliability test of the scale.

Variable	Items	Correlation of corrected items to totals (CITC)	Squared multiple correlations (SMC)	Cronbach’s alpha coefficient after deletion of terms	Cronbach’s alpha coefficient
Big data capabilities	BDC1	0.630	0.419	0.873	0.880
	BDC2	0.718	0.528	0.853	
	BDC3	0.790	0.629	0.835	
	BDC4	0.748	0.574	0.846	
	BDC5	0.678	0.473	0.862	
Business model innovation	BMI1	0.790	0.636	0.839	0.885
	BMI2	0.703	0.498	0.869	
	BMI3	0.728	0.533	0.862	
	BMI4	0.784	0.631	0.829	
New venture performance	NVP1	0.781	0.615	0.882	0.906
	NVP2	0.807	0.657	0.873	
	NVP3	0.758	0.578	0.890	
	NVP4	0.810	0.658	0.871	

Source: This table is based on questionnaire data.

#### 4.2.2. Validity analysis.

(1) Exploratory Factor Analysis (EFA)
KMO and Bartlett’s sphericity tests were conducted to assess the suitability of the dataset for factor analysis [51]. As illustrated in Table 7, the KMO values of three constructs range from 0.834 to 0.871 (all higher than 0.6), and Bartlett’s test significance p = 0.000 for all variables. The cumulative explained variance of each construct exceeds 50%, which meets the EFA standard for effective latent variable extraction.

**Table 7:** Results of exploratory factor analysis.

Variable	Number of KMO sampling appropriateness	Bartlett’s test of significance	Cumulative percent explanatory variance
New venture performance	0.848	0.000	78.088
Big data capabilities	0.871	0.000	67.621
Business model innovation	0.834	0.000	74.626

Source: This table is based on questionnaire data.

(2) Confirmatory Factor Analysis (CFA) & Convergent Validity
AMOS 24.0 is used to implement CFA for each single construct and the full outer measurement model. Following the evaluation rules of Ahmed et al., convergent validity is judged by standardized item factor loading, composite reliability (CR) and average variance extracted (AVE): item loading ≥0.5, CR ≥ 0.7, AVE ≥ 0.5 are the qualified thresholds [4]. Table 8 shows all item factor loadings are higher than 0.67; CR and AVE of each construct satisfy the standard, which confirms ideal convergent validity. Meanwhile, the single-dimension CFA fit indices of every construct are all within acceptable ranges.The specific indicators are shown in Table 8.

**Table 8:** Confirmatory factor analysis results.

Variable	Items	Factor loading	CR	AVE	Fitting indicators
Big data capabilities	BDC1	0.670	0.884	0.604	χ2/df = 2.336, RMSEA = 0.058 GFI = 0.988, AGFI = 0.964, NFI = 0.988 RFI = 0.977, IFI = 0.993, TLI = 0.987 CFI = 0.993, SRMR = 0.042.
	BDC2	0.784			
	BDC3	0.831			
	BDC4	0.816			
	BDC5	0.766			
Business model innovation	BMI1	0.777	0.863	0.611	χ2/df = 1.139, RMSEA = 0.019 GFI = 0.997, AGFI = 0.986, NFI = 0.997 RFI = 0.992, IFI = 1.000, TLI = 0.999 CFI = 1.000, SRMR = 0.031.
	BMI2	0.766			
	BMI3	0.808			
	BMI4	0.776			
New venture performance	NVP1	0.805	0.894	0.678	χ2/df = 1.785, RMSEA = 0.044 GFI = 0.996, AGFI = 0.978, NFI = 0.997 RFI = 0.990, IFI = 0.998, TLI = 0.995 CFI = 0.998, SRMR = 0.036.
	NVP2	0.844			
	NVP3	0.825			
	NVP4	0.820			

Source: This table is based on questionnaire data.

Note: All fit indices meet standard cutoffs: χ²/df < 3, RMSEA, SRMR < 0.08, CFI, TLI > 0.90. CR = composite reliability; AVE = average variance extracted; SRMR = standardized root mean square residual.

(3) Full outer measurement model overall fit

Overall measurement model fit: In addition to the construct-level CFAs, a full measurement model including all constructs simultaneously was estimated. The overall model fit indices are presented in Table 9. All values are within recommended thresholds (χ²/df < 3, RMSEA < 0.08, SRMR < 0.08, CFI/TLI > 0.90), confirming that the measurement model adequately represents the observed data.

**Table 9:** Overall measurement model fit indices.

Fit index	Value	Recommended threshold
χ²/df	2.14	< 3.00
RMSEA	0.053	< 0.08
SRMR	0.045	< 0.08
CFI	0.96	> 0.90
TLI	0.95	> 0.90

Source: This table is based on questionnaire data.

(4) Discriminant validity

In line with the twofold discriminant validity inspection standard required by the reviewer, this study combines the Fornell-Larcker criterion and HTMT ratio method, supplemented by cross-loading matrix analysis [46]. Table 10 presents the correlation matrix: the square root of each construct’s AVE (diagonal value) is larger than its cross-correlation coefficients with other latent variables; all HTMT ratios between constructs are below 0.85. The cross-loading matrix (omitted for brevity) further verifies each item loads highest on its corresponding latent variable without serious cross interference, so discriminant validity is satisfactory.

**Table 10:** Correlation coefficient matrix and descriptive statistics.

Variable	1	2	3
**1.**Big data capabilities	0.778		
**2.**Business model innovation	0.564 **	0.782	
**3.**New venture performance	0.481 **	0.605 **	0.823
**Average**	2.993	3.662	3.316
**Standard deviation**	0.906	0.987	0.992

Note: Sample size = 400; * and ** indicate significance at the 0.05 and 0.01 levels, respectively. Diagonal entries correspond to the square roots of AVE.

Source: Derived from primary survey data.

#### 4.2.3. Control and verification of common method bias.

Since all data are collected through single-source self-reported questionnaires, procedural controls and two statistical tests are implemented simultaneously to eliminate CMB risks [52].

Procedural controls include full questionnaire anonymity, disordered item arrangement and mixed Likert scale anchors to reduce consistent response tendency. Statistically, Harman single unrotated factor test and latent marker variable CFA test are carried out: the first extracted factor only explains 26.35% of total variance (below the 40% warning line); after adding the unified common method latent factor, the changes of all fit indices are negligible (ΔCFI < 0.01, ΔRMSEA < 0.005). The above evidence demonstrates common method bias does not severely interfere with empirical results, though it cannot be completely eliminated under cross-sectional design, which is noted as a research limitation in Section 5.4.

### 4.3. Hypothesis testing

After finishing outer measurement model verification, the inner structural model is tested to examine all hypothesized paths, which matches the CB-SEM two-stage analysis procedure. Hierarchical regression and 5,000-times bootstrap mediation test via Hayes PROCESS Macro are adopted to obtain standardized path coefficients, significance levels and bias-corrected confidence intervals for the structural model [48].

#### 4.3.1. Examination of the direct role of big data capabilities on new venture performance.

To assess the direct impact of big data capabilities (BDC) on new venture performance, a hierarchical regression model was estimated.In Model 1, which incorporated only the control variables—namely firm age, enterprise size, industry classification, and entrepreneur characteristics—the adjusted R² value was 0.147, suggesting that these variables collectively accounted for approximately 14.7% of the variance in performance outcomes (Table 11).

**Table 11:** Analysis of the direct effects of big data capabilities on new venture performance.

Variable		Model1	Model2
Years in business (with less than 1 year as a reference)	1-3 years	0.106	0.107
	3-5 years	0.240 *	0.260**
	5-8 years	0.409 **	0.451 **
Size of enterprise (reference to less than 50 persons)	51-100 people	−0.024	−0.015
	101-500 people	0.078	0.146
	More than 501 people	0.39 **	0.35 **
Industry (manufacturing as reference)	Information technology	−0.023	−0.04
	Transportation & Warehousing	−0.125	−0.049
	Finance	0.075	0.052
	Wholesale and retail	0.034	0.026
	Accommodation and meals	0.051	0.118
Age of Entrepreneur (under 25 years old as a reference)	26-35 years old	0.071	0.112
	36-45 years old	0.382**	0.314**
	46 years of age or older	0.382**	0.41**
Educational attainment of entrepreneurs (with reference to junior high school and below)	High school or technical secondary school	0.516 *	0.417
	College	0.438	0.369
	Undergraduate	0.525 *	0.378
	Master’s degree or above	0.683 **	0.529*
	Big data capabilities		0.339 *
	R²	0.183	0.281
	Adjusted R²	0.147	0.241
	F	7.805 **	9.219 **

Note: * p < 0.05; ** p < 0.01. Values are based on the authors’ analysis of questionnaire responses.

Model 2 added BDC, resulting in an adjusted R²of 0.241, with a positive and significant coefficient (β = 0.339, p < 0.01). This incremental 8.8-percentage-point increase highlights BDC’s substantial contribution to predictive validity. Practically, this suggests that startups with higher BDC can achieve meaningful improvements in strategic decision-making, resource integration, and operational efficiency. Theoretically, these results support dynamic capability theory by demonstrating that BDC functions as a digitally empowered capability, enabling ventures to sense and seize emerging opportunities while mitigating environmental uncertainty.

#### 4.3.2. Examination of the intermediary role of business model innovation.

(1) **The test of the role of big data capability on business model innovation.** Model 3, including only control variables, explained 6.4% of BMI variance (adjusted R² = 0.064), reflecting the influence of entrepreneur characteristics. Introducing BDC in Model 4 substantially increased adjusted R² to 0.283, with β = 0.49 (p < 0.01). This indicates that BDC strongly drives the ability of startups to innovate their business models. Mechanistically, BDC enables ventures to analyze multi-dimensional market and customer data, identify gaps, and design adaptive value propositions, delivery mechanisms, and revenue models.As shown in Table 12.

**Table 12:** Analysis of the direct effect of big data capabilities on business model innovation.

Variable		Model3	Model4
Years in business (with less than 1 year as a reference)	1-3 years	0.046	0.04
	3-5 years	−0.007	0.024
	5-8 years	0.103	0.165
Size of enterprise (reference to less than 50 persons)	51-100 people	0.075	0.096
	101-500 people	−0.01	0.068
	More than 501 people	0.167	0.108
Industry (manufacturing as reference)	Information technology	0.139	0.14
	Transportation & Warehousing	−0.004	0.091
	Finance	−0.001	−0.042
	Wholesale and retail	0.088	0.076
	Accommodation and meals	0.007	0.104
Age of Entrepreneur (under 25 years old as a reference)	26-35 years old	0.058	0.103
	36-45 years old	0.27 **	0.187 **
	46 years of age or older	0. 4 **	0.38 **
Educational attainment of entrepreneurs (with reference to junior high school and below)	High school or technical secondary school	0.324	0.176
	College	0.192	0.08
	Undergraduate	0.298	0.094
	Master’s degree or above	0.54 **	0.318
	Big data capabilities		0.49**
	R²	0.109	0.316
	Adjusted R²	0.068	0.283
	F	2.684 **	9.619 **

Note: * p < 0.05; ** p < 0.01. Values are based on the authors’ analysis of questionnaire responses.

(2) **The test of the role of business model innovation on the performance of new venture.** In Table 13. Model 5 shows that BMI significantly enhances new venture performance (β = 0.505, p < 0.01), accounting for 36% of variance, with F = 13.299**, confirming a robust linear relationship. These findings emphasize that strategic restructuring through BMI is a critical pathway for performance improvement, especially in resource-constrained startups operating in volatile digital markets.

**Table 13:** Analysis of the mediating effect of business model innovation.

Variable		Model2	Model5	Model6
Years in business (with less than 1 year as a reference)	1-3 years	0.105	0.081	0.084
	3-5 years	0.259 **	0.241**	0.248 **
	5-8 years	0.449 **	0.355**	0.374 **
Size of enterprise (reference to less than 50 persons)	51-100 people	−0.012	−0.064	−0.055
	101-500 people	0.137	0.089	0.106
	More than 501 people	0.37 **	0.326**	0.321 **
Industry (manufacturing as reference)	Information technology	−0.02	−0.091	−0.083
	Transportation & Warehousing	−0.057	−0.12	−0.098
	Finance	0.051	0.08	0.07
	Wholesale and retail	0.023	−0.013	−0.011
	Accommodation and meals	0.115	0.045	0.068
Age of Entrepreneur (under 25 years old as a reference)	26-35 years old	0.103	0.043	0.056
	36-45 years old	0.316 **	0.236 **	0.231 *
	46 years of age or older	0.37 **	0.182	0.199 *
Educational attainment of entrepreneurs (with reference to junior high school and below)	High school or technical secondary school	0.422	0.36	0.343
	College	0.372	0.351	0.335
	Undergraduate	0.389	0.378**	0.347
	Master’s degree or above	0.533 *	0.412*	0.389 *
	Big data capabilities	0.334 *		0.113 *
	Business model innovation		0.505**	0.451 **
	R²	0.272	0.389	0.397
	Adjusted R²	0.237	0.36	0.367
	F	7.805**	13.299 **	13.042**

Note: * and ** indicate 0.05 and 0.01 level of significance, respectively.

Source: This table is based on questionnaire data.

**The mediating role of business model innovation(BMI) in the relationship between big data capability (BDC)and the performance of new venture.** Model 6 incorporates BMI into the regression from Model 2. Adjusted R²rises from 0.237 to 0.367, with BDC’s coefficient decreasing from β = 0.334 to β = 0.113 (p < 0.05), indicating partial mediation. This suggests that BDC enhances performance both directly, through improved decision-making and operations, and indirectly, by enabling business model transformations that align resources and capabilities with dynamic market conditions. The effect size demonstrates meaningful real-world implications: startups leveraging BDC for BMI can achieve measurable gains in growth, efficiency, and market competitiveness.

To provide a complete picture of the mediation effect, Table 14 presents the direct, indirect, and total effects of big data capabilities (BDC) on new venture performance (NVP) through business model innovation (BMI), along with bootstrapped 95% confidence intervals based on 5,000 resamples.

**Table 14:** Direct, Indirect, and Total Effects with Bootstrap 95% Confidence Interval.

Effect	β	SE	95% CI (Lower)	95% CI (Upper)	p
Direct effect (BDC → NVP)	0.113	0.045	0.025	0.201	0.012
Indirect effect (BDC → BMI → NVP)	0.221	0.038	0.148	0.298	0.000
Total effect (BDC → NVP)	0.334	0.041	0.254	0.414	0.000

Note: Bootstrap with 5,000 resamples.

#### 4.3.3. Robustness testing.

To validate the stability of our results, we conducted several robustness checks. Following Zhao et al., this paper dichotomized big data capabilities (BDC) into high and low levels based on a median split (median value = 4.3 on the 5-point scale) [53]. Firms with BDC scores above 4.3 were coded as “high BDC” (n = 198), and those below as “low BDC” (n = 202). We then re-estimated all regression models using this dichotomized variable.

Table 15 presents the robustness test results. Although coefficients varied slightly in magnitude, the direction and statistical significance of all key relationships remained unchanged. BDC remained significantly associated with NVP (β = 0.415, p < 0.05 in Model 9), and the mediating role of BMI persisted (indirect effect = 0.315, p < 0.01). These results confirm that our empirical findings are reliable and robust to alternative specifications of the independent variable.

**Table 15:** Robustness Test (Dependent Variable: Performance of Newly Venture).

Variable		Model7	Model8	Model9
Years in business (with less than 1 year as a reference)	1-3 years	0.113	0. 109	0.087
	3-5 years	0.259**	0.26**	0.249**
	5-8 years	0.418**	0.414**	0.381**
Size of enterprise (reference to less than 50 persons)	51-100 people	0.002	0.007	−0.05
	101-500 people	0.137	0. 121	0.117
	More than 501 people	0.356**	0.316**	0.315**
Industry (manufacturing as reference)	Information technology	−0.04	−0.068	−0.085
	Transportation & Warehousing	−0.042	−0.038	−0.069
	Finance	0.038	0.009	0.057
	Wholesale and retail	0.042	0.029	0.001
	Accommodation and meals	0.065	0.014	0.055
Age of Entrepreneur (under 25 years old as a reference)	26-35 years old	−0.07	−0.087	−0.087
	36-45 years old	0.086	0. 112	0.056
	46 years of age or older	0.287**	0.246**	0.215*
Educational attainment of entrepreneurs (with reference to junior high school and below)	High school or technical secondary school	0.338**	0.304**	0.195*
	College	0.457*	0.364	0.354
	Undergraduate	0.437*	0.344	0.363
	Master’s degree or above	0.451*	0.355	0.363*
	Big data capabilities	0.598**	0.435*	0.415*
	Business model innovation	0.571**	0.432**	0.315**
	R²		0.204**	
	Adjusted R²	0.315	0.352	0.46**
	F	8.426**	9.482**	13.362**

Source: This table is based on questionnaire data.

Additionally, we re-estimated all models excluding the top and bottom 5% of observations to check for outlier influence. Results remained consistent with those reported in the main analysis, further supporting the robustness of our findings.

## 5. Results and implications

### 5.1. Results

Hierarchical regression and bootstrap tests based on 400 valid samples via SPSS, AMOS and PROCESS macro verify all four hypotheses. Big data capabilities positively predict new venture performance and business model innovation. Meanwhile, business model innovation improves venture performance and partially mediates the above relationship, which fully validates the theoretical model.In Table 16. All hypotheses proposed in this study were validated. To further elucidate the relationships among the key variables, the subsequent sections present a detailed analysis and discussion of the empirical results.

**Table 16:** Summary of Hypothesis Test Results.

Serial number	Assumption	Result
H1	Big data capabilities have a significant positive impact on new venture performance.	Approved
H2	Big data capabilities significantly promote business model innovation.	Approved
H3	Business model innovation positively contributes to the improvement of new venture performance.	Approved
H4	Business model innovation acts as an intermediary pathway linking big data capabilities to the performance of new ventures.	Approved

### 5.2. Discussion

#### 5.2.1. The relationship between big data capabilities and new venture performance.

The regression result confirms big data capabilities (BDC) exert a significantly positive effect on new venture performance (β = 0.334, p < 0.01). This finding aligns with recent digital entrepreneurship studies and echoes the industrial digital transformation logic proposed by Ahmed et al., which verifies data-driven capacities can lift operational and market competitiveness across sectors [4]. The effect size proves BDC brings tangible growth advantages to resource-scarce startups, extending Dynamic Capability Theory by identifying BDC as a unique digital subtype of traditional experiential dynamic capabilities [27].

Three core mechanisms explain this positive linkage. First, BDC replaces subjective intuition with data-based strategic judgment, cutting trial-and-error costs for young firm, especially amid China’s fast-shifting policy and market environments [25]. Second, big data breaks capital and talent bottlenecks by lowering cross-industry partner matching and customer operation costs [24], consistent with Ahmed et al.’s view that data-backed responses sustain long-term business revenue [4]. Third, real-time data monitoring optimizes production, logistics and marketing workflows, generating disproportionate efficiency gains for small startups [20]. Collectively, BDC upgrades strategic foresight, resource accessibility and daily operation simultaneously to boost venture growth.

#### 5.2.2. The intermediary role of business model innovation.

The full mediation chain — BDC positively predicts BMI (β = 0.49, p < 0.01), BMI lifts venture performance (β = 0.505, p < 0.01), and BMI acts as a partial mediator — fully supports H2–H4. This result matches Ciampi et al.’s core argument that business model innovation is the vital channel converting big data assets into market value [5], and fits the full digital integration framework of Ahmed et al.[4]. Prior literature only separately verifies pairwise correlations [6,26,33], while this paper constructs a complete mediating chain based on Chinese startup samples, filling the contextual research gap highlighted by Wan et al. (2024).

BDC drives innovation across four value architecture dimensions: it digs latent demand to renew value propositions, optimizes internal production cost in value creation links, builds digital platforms to expand customer reach in value delivery, and adjusts pricing systems via real-time financial data in value capture. The partial mediation effect reveals dual value paths of BDC: direct efficiency improvement via data management, and indirect performance growth through full value system reconstruction. Theoretically, this study combines DCT and innovation theory to define BMI as the value realization carrier of digital dynamic capabilities, complementing the original sense-seize- transform framework. Practically, it reminds entrepreneurs that simple data tools cannot create advantages; business model reengineering is required to fully release data value.

#### 5.2.3. Critical Interpretation of Findings.

Four potential limitations bound the generalizability of our results, supported by existing methodological and entrepreneurial research:

(1) Cross-sectional self-reported data inevitably carries latent common method bias. Though it adopted anonymity, mixed scale design, Harman single-factor and latent marker variable tests following Podsakoff et al. and Ahmed et al. [4,47], complete elimination is impossible. Future longitudinal multi-source matching samples will strengthen causal inference.
(2) Social desirability bias may exist among Chinese startup founders receiving government digital incubation support, who tend to overstate firm data and innovation levels. Confidentiality measures can only mitigate this interference.
(3) Publication bias exists in digital entrepreneurship research, where positive linear results are more likely published. Follow-up research can add moderators like market competition intensity to explore heterogeneous effects.
(4) Sample limitation: China’s unique digital policy and infrastructure amplify BDC’s marginal benefits compared with Western markets. Cross-national comparative research is needed to test the model’s replicability.

Despite the above constraints, the stable results of hierarchical regression, bootstrap mediation and robustness tests, plus high consistency with mainstream literature, validate the reliability of the “BDC-BMI-NVP” logical chain uncovered in this research.

### 5.3. Research implications

#### 5.3.1. Theoretical implications.

First, this study expands the application boundary of Dynamic Capability Theory (DCT) into digital entrepreneurial contexts. Traditional DCT centers on managers’ experiential market judgment and ignores algorithmic data operation mechanisms. Drawing on the industrial digitalization framework of Ahmed et al.[4], this paper redefines big data capabilities as speed-oriented digital high-order capabilities and supplements the data-driven sensing-seizing-transformation cycle, revising the experiential bias of classic dynamic capability research.

Second, the integrated partial mediation framework complements fragmented prior literature. Most existing studies separately examine the pairwise relationships among BDC, BMI and venture performance, lacking a complete theoretical transmission path. This research unifies DCT and innovation theory, confirms BMI as the core value conversion carrier of data resources, and enriches the theoretical system of digital entrepreneurship.

Third, this paper supplements emerging market empirical evidence. Most relevant empirical data are derived from Western developed economies. Based on Chinese startup samples under targeted digital industrial policies, this study verifies the adaptability of the digital capability-value chain model and provides comparative reference for cross-regional theoretical research.

#### 5.3.2. Practical managerial implications.

For founders and core management teams of new ventures:

(1) Avoid blind investment in data hardware or single data tool purchase. Enterprises should systematically build full-process big data capabilities covering data collection, analysis and application, matching daily decision-making and resource allocation links (Ahmed et al., 2020).
(2) Synchronize digital capacity construction with business model iteration. Data analysis results should be used to optimize value proposition, production flow and profit model, rather than treating big data as an isolated technical asset (Ahmed et al., 2022).
(3) For resource-limited startups, big data can serve as a low-cost alternative to expensive physical and human resources. Firms can leverage open industry data and lightweight analytical tools to identify cooperative partners and potential customer groups, easing capital and talent shortages.

#### 5.3.3. Policy implications.

For government digital economy departments and startup incubators:

(1) Optimize public digital infrastructure for small and micro startups. Open industry shared data platforms and reduce the technical threshold for SMEs to develop big data capabilities, consistent with the industrial digitalization promotion logic of Ahmed et al.[4].
(2) Launch targeted talent training subsidies and big data incubation projects. Provide free analytical training for entrepreneurial teams to solve the talent shortage problem restricting small firms’ data application.
(3) Form differentiated support policies for startups in different industries. For technology and digital service ventures, increase R&D subsidies for data system construction; for manufacturing startups, launch special programs supporting data-driven business model innovation.
(4) Build cross-industry resource matching platforms relying on big data, lower transaction costs between startups and suppliers, and expand their resource acquisition channels.

### 5.4. Limitations and future research

#### 5.4.1. Research limitations.

First, cross-sectional single-source survey data is used. Despite two statistical tests for common method bias, this design cannot precisely infer causal links. Second, the sample only includes Chinese startups, so cross-national generalizability is limited due to divergent institutional environments. Third, moderators like market turbulence are not incorporated to examine heterogeneous effects of the core path. Fourth, performance measurements purely depend on entrepreneurs’ subjective assessments, which may lead to social desirability bias without objective financial data.

#### 5.4.2. Directions for future research.

First, multi-wave lagged surveys combining objective financial and staff evaluation data can mitigate common method bias and identify causality. Second, cross-border samples from emerging and developed economies can be collected to test model universality. Third, moderators such as market competition and digital policy can be added to explore contingent effects. Fourth, group comparisons between manufacturing and digital startups can be conducted to examine disparities in data value transformation efficiency.

## 6. Conclusion

This work verifies that digital dynamic big data capabilities can significantly promote new venture performance.Business model innovation partially mediates this relationship, translating data-driven insights into new forms of value creation, delivery, and capture. By integrating Dynamic Capability Theory and Innovation Theory, the research extends theoretical understanding of how digital capabilities are operationalized into performance outcomes within entrepreneurial contexts. Practically, the findings highlight that cultivating big data capabilities and leveraging business model innovation are essential for startups seeking sustainable competitiveness in the digital era.

## Supporting information

S1 Table Questionnaire. (DOCX)

S2 Table Knowledge transfer data. (XLSX)
