# Supplementary material for: The impact of big data capabilities and business model innovation on new venture performance
Source: PLoS One. 2026 Sep 17;21(9):e0358363. doi: 10.1371/journal.pone.0358363 (PMC13585229; doi:10.1371/journal.pone.0358363)
Supplement: Supplementary file 1 [file pone.0358363.s001.docx]

**Appendix A: Full Survey Instrument**

**Survey on Big Data Capabilities, Business Model Innovation, and Firm Performance**

Dear Participant,

Thank you for participating in this academic survey. This research examines how big data capabilities, business model innovation, and entrepreneurial performance are interrelated. All responses will be used exclusively for scholarly research and will be kept strictly confidential.Please complete the questionnaire based on your organization's actual circumstances. We greatly appreciate your time and support!

**Part 1: Basic Information**

1. Your gender: □ Male □ Female

2. Your age: □ Under 25 □ 26-35 □ 36-45 □ 46 or above

3. Your highest education level: □ High school or below □ Associate degree □ Bachelor's degree □ Master's degree or above

4. Years since firm founding: □ <1 year □ 1-3 years □ 3-5 years □ 5-8 years

5. Number of employees: □ <10 □ 10-50 □ 51-100 □ 101-500 □ >500

6. Industry sector: □ Manufacturing □ Technology □ Services □ Other

**Part 2: Big Data Capabilities (BDC)**

*(5-point Likert scale: 1 = Strongly Disagree, 5 = Strongly Agree)*

| **Item Code** | **Item Wording** |
| --- | --- |
| BDC1 | Enterprises can obtain various data from internal and external channels in a timely manner. |
| BDC2 | Firms are able to engage in ongoing learning and regularly upgrade their big data technologies. |
| BDC3 | Organizations are capable of attracting and developing professionals with expertise in big data technologies. |
| BDC4 | Firms possess the capability to handle and manage large volumes of data efficiently. |
| BDC5 | Enterprises can analyze various types of unstructured data (images, sound, video, etc.) in real time. |

**Part 3: Business Model Innovation (BMI)**

*(5-point Likert scale: 1 = Strongly Disagree, 5 = Strongly Agree)*

| **Item Code** | **Item Wording** |
| --- | --- |
| BMI1 | The products/services launched by the company are innovative and can meet customer needs. |
| BMI2 | The company can accurately identify and expand new customer groups. |
| BMI3 | The firm has embedded innovation within its key operational areas, including design, logistics, and marketing functions. |
| BMI4 | The company effectively acquires new resources and capabilities (technology, talent, information technology systems, etc.) necessary to conduct business. |

**Part 4: New Venture Performance (NVP)**

*(5-point Likert scale: 1 = Strongly Disagree, 5 = Strongly Agree)*

| **Item Code** | **Item Wording** |
| --- | --- |
| NVP1 | The firm reports greater annual sales revenue relative to its industry peers. |
| NVP2 | The company achieves superior profit margins in comparison to its competitors. |
| NVP3 | The company demonstrates a higher return on investment than its competitors. |
| NVP4 | The firm shows a greater return on assets relative to its competitors. |
